# Discover the network mechanisms underlying the connections between aging and age-related diseases

**DOI:** 10.1038/srep32566

**Published:** 2016-09-01

**Authors:** Jialiang Yang, Tao Huang, Won-min Song, Francesca Petralia, Charles V. Mobbs, Bin Zhang, Yong Zhao, Eric E. Schadt, Jun Zhu, Zhidong Tu

**Affiliations:** 1 Institute of Genomics and Multiscale Biology, Icahn School of Medicine at Mount Sinai, NY, 10029, USA; 2 Department of Genetics and Genomic Sciences, Icahn School of Medicine at Mount Sinai, NY, 10029, USA; 3 Department of Neuroscience, Icahn School of Medicine at Mount Sinai, NY, 10029, USA; 4 Department of Geriatrics and Palliative Medicine, Icahn School of Medicine at Mount Sinai, NY, 10029, USA

## Abstract

Although our knowledge of aging has greatly expanded in the past decades, it remains elusive why and how aging contributes to the development of age-related diseases (ARDs). In particular, a global mechanistic understanding of the connections between aging and ARDs is yet to be established. We rely on a network modelling named “GeroNet” to study the connections between aging and more than a hundred diseases. By evaluating topological connections between aging genes and disease genes in over three thousand subnetworks corresponding to various biological processes, we show that aging has stronger connections with ARD genes compared to non-ARD genes in subnetworks corresponding to “response to decreased oxygen levels”, “insulin signalling pathway”, “cell cycle”, etc. Based on subnetwork connectivity, we can correctly “predict” if a disease is age-related and prioritize the biological processes that are involved in connecting to multiple ARDs. Using Alzheimer’s disease (AD) as an example, GeroNet identifies meaningful genes that may play key roles in connecting aging and ARDs. The top modules identified by GeroNet in AD significantly overlap with modules identified from a large scale AD brain gene expression experiment, supporting that GeroNet indeed reveals the underlying biological processes involved in the disease.

Aging is a major risk factor for age-related diseases (ARDs). For example, the risks of developing certain cancers, cardiovascular disease, Alzheimer’s disease (AD), Parkinson’s disease, and type 2 diabetes (T2D), all increase dramatically with age^1^,^2^. As human life expectancy expands, the number of patients having ARDs has increased rapidly and will continue to rise in the near future, posing a serious challenge to the health care system globally.

As we search for the ultimate cause of aging and ARDs^3^, an increasing number of mechanisms have been proposed for their roles in linking aging and ARDs. For example, genomic instability and decreased capacity for DNA repair are commonly seen in both cancer and aging^4^; telomere length and telomerase activity are reported to play important roles in aging and diseases like Alzheimer’s dementia^5^; mitochondrial dysfunction is a hallmark of aging and ARDs including cancer and cardiovascular diseases^6^,^7^; chronic inflammation is known to associate with aging and is likely to contribute to ARDs like diabetes^8^, cardiovascular diseases^9^, and neurodegenerative diseases^10^. However, most existing studies either focused on specific diseases, or specific aging mechanisms such as sirtuins^11^ and insulin/IGF-1^12^. A systems understanding of the molecular mechanisms underlying the connections between aging and ARDs is yet to be established and multiple key questions remain to be answered. For example, why do diseases like AD and T2D mainly manifest themselves at old ages but remain silent prior to that? What pathways are involved that contribute to the development of ARDs? Are some pathways more important than others, and how disease specific are they?

Several network-based analyses have been reported to study the connection between aging and ARDs. For example, Wolfson *et al*. built protein-protein interaction (PPI) networks of cancer, longevity, atherosclerosis, T2D, and Alzheimer’s disease and defined a common signature of 588 proteins that were shared by these networks. This common PPI network of longevity and major ARDs is significantly enriched for signaling proteins and interactome hubs^13^. In a related work, Tacutu *et al*. found that cellular senescence (CS) was closely interconnected with aging, longevity, and ARDs, either by sharing common genes and regulators or by protein-protein interactions, and eventually by common signaling pathways^14^. Both works highlighted the close interactions between aging or senescence genes with ARD genes. However, they only considered a very limited number of ARDs; therefore it remains unclear if their findings can be generalized to other diseases. In another work, Wang *et al*. constructed a network connecting aging genes and disease genes from a large number of categories. They found that human aging genes were topologically closer to disease genes than by random chance; their analysis also indicated that diseases could be categorized into two types according to their relationships with aging. Type 1 diseases have their genes significantly close to aging genes, while type 2 diseases do not^15^. This work provided an intriguing insight to the aging-disease connections and indicated that diseases could have varied degrees of connection with aging. However, all these studies only considered a global human PPI network, which provided poor specificity for revealing the disease-aging connections at single pathway level. In addition, all these works only considered direct interactions between aging and diseases (e.g., overlap genes or direct physical interactions in PPI network); the ignorance of indirect interactions (i.e., genes interacting only through other genes) may lead to overlook of some important connections between aging and diseases.

In this study, we develop a network approach named GeroNet to comprehensively evaluate both direct and indirect connections between aging and 149 diseases in more than three thousand subnetworks. Using AD as an example, we show that meaningful biological processes involved in the disease and key genes that potentially mediate disease-aging connections could be identified. The top subnetworks identified by GeroNet highly overlap with the top modules identified from a recent AD brain gene expression experiment^16^, suggesting that GeroNet indeed reveals the underlying biological processes involved in the disease.

## Results

**Figure 1:**
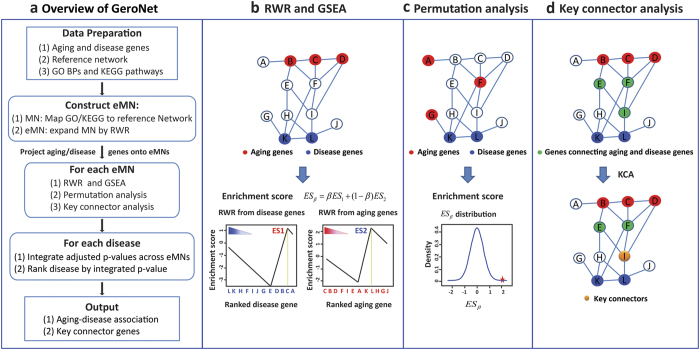
An overview of GeroNet and its main algorithms. (**a**) An overview of GeroNet. RWR: random walk with restart, MN and eMN: modularized network and expanded modularized network, and GSEA: gene set enrichment analysis. (**b**) Starting from either aging genes (red nodes) or disease genes (blue nodes), an RWR is performed to calculate for each node, its steady state probability of being visited in the subnetwork. A method similar to GSEA is applied to calculate a running sum estimating the reachability between aging genes and disease genes. Two enrichment scores (*ES*_1_ and *ES*_2_, corresponding to starting the RWR from either aging genes or disease genes, respectively) are combined as 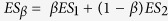. (**c**) A permutation test is used to evaluate the significance level of *ES*_*β*_. (**d**) Key connector genes (orange nodes) are identified from common genes between expanded disease and aging genes.

**Figure 2:**
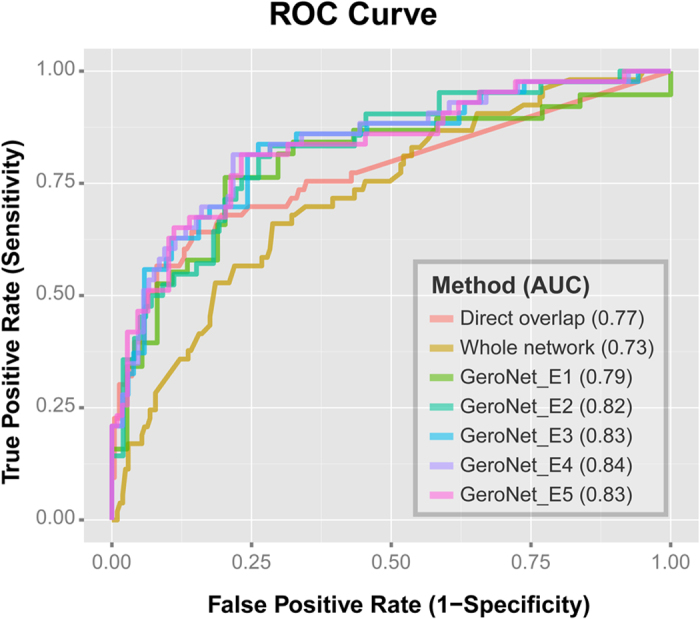
Comparison of different methods based on AUC of ROC. It is of note that we denote GeroNet with different expansion fold N as GeroNet_E*N* (for ***N*** = 1, 2, …, 5). “Direct Overlap” indicates the ranking method by Jaccard indices between aging and disease/trait genes, and “Whole network” refers to the use of whole PPI network for GeroNet input.

**Figure 3:**
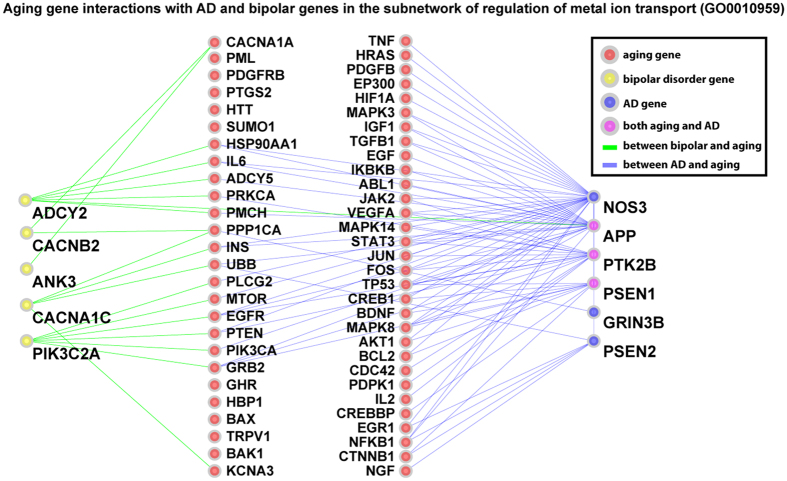
Interactions between aging and AD/BD genes in the subnetwork of regulation of metal ion transport. Significantly more interactions are seen between AD and aging genes, compared to the interactions between BD and aging genes. Red node: aging gene; yellow node: BD gene; blue node: AD gene; and purple node: aging and AD gene. A blue edge connects aging and AD genes, whereas a green edge connects aging and BD genes.

An overview of the GeroNet approach is illustrated in Fig. 1a. GeroNet takes a list of aging genes, lists of genes corresponding to various disease categories, a reference human PPI network, and a comprehensive list of GO Biological Processes (BPs)/KEGG pathways as input. It performs the following steps: (1) Genes in each GO BP or KEGG pathway are mapped to the reference PPI network to define a modularized network (MN). Each MN is expanded by a Random Walk with Restart (RWR) procedure to form an expanded modularized network (eMN) to ensure it is of a certain size; (2) Aging genes and genes for a particular disease are mapped to each eMN; The mutual reachability between the two mapped gene sets is estimated using RWR and gene set enrichment analysis (GSEA); The significance of the mutual reachability is evaluated by a permutation analysis, in which the aging genes are randomly permuted, and the significance p-value is adjusted for multiple testing (Fig. 2b); (3) A key connector analysis (KCA) is applied to significant eMNs to identify the top genes connecting aging and disease genes; (4) The association between aging and the disease is evaluated by the geometric mean of adjusted p-values for the reachability of their genes across all eMNs; (5) The whole procedure is repeated for every disease under investigation and diseases are ranked according to the geometric means of the p-values, where a disease with smaller p-value is considered to be more age-related. Detailed information is provided in the Methods section.

### Collection of aging and disease genes, reference PPI network, GO BPs/KEGG pathways, and ARD classification

We used GenAge human aging genes as our input of aging genes^17^. GenAge collected its candidate human aging genes through manual curation of more than 2,200 literatures. It contains genes directly linked to human aging, the homologous genes with the strongest evidence from model organisms, and genes meeting several other criteria supporting their functional roles in aging. The reference PPI network was obtained from Human Protein Reference Database (HPRD)^18^ and Search Tool for the Retrieval of Interacting Genes/Proteins (STRING, Version 10)^19^. Six confidence levels (based on confidence score cut-offs from 400 to 900 with step size of 100) of PPIs provided by STRING were considered. We considered 19,540 GO BPs and 294 KEGG pathways to define modularized networks. 277 disease or trait categories were obtained by merging genes from NIH GWAS Catalogue and Online Mendelian Inheritance in Man (OMIM) (see Methods). The number of disease genes for each category ranges from 5 to 431 and a full list of our disease genes is provided in Dataset S1. We annotated disease categories as being either age-related (e.g., Alzheimer’s disease) or non-age related (e.g., type 1 diabetes) using a literature based text-mining followed by manual curation. 53 diseases/traits were annotated as ARDs and the rest were considered as non-ARDs (Table S2). The full information of this annotation is provided in Dataset S2.

### Model selection and parameter optimization

There are multiple ways of modelling the connection between aging and disease genes in a network. For example, we can consider direct overlap (e.g., using Jaccard index) to estimate the overlap between aging and disease genes. Alternatively, we can use GeroNet but apply it to whole PPI network instead of modularized networks. Also for GeroNet, parameters need to be set and optimized (i.e., different network inputs, the expansion fold *N* and coefficient *β* in equation (4) in Methods). To compare models and select model parameters, we rely on the accuracy of classifying diseases into ARDs vs. non-ARDs by each method. Ideally, a good method would rank ARDs on the top of disease list and put non-ARDs to the bottom based on its scoring function. To quantify the performance, we calculated the Area Under the Receiver Operating Characteristic curve (AUROC or simply AUC) for each model, a commonly used statistics to characterize the overall performance of a predictive model. The results for GeroNet, whole network, and direct overlap with various network inputs and parameters are plotted in Fig. 2. For different network inputs, we only plotted the ones that delivered the best AUROC. Additional results are listed in Table S3.

As can be seen in Fig. 2, GeroNet outperformed direct overlap and whole network methods. We also tested 5 values of expansion fold *N* (i.e., 1, 2, 3, 4, and 5) and denoted the corresponding methods by GeroNet_EN. The expansion fold of modularized networks has minor effect on AUCs, and four-fold expansion GeroNet_E4 performed the best with AUROC of 0.84. For different input PPI networks, GeroNet_E4 performed the best on STRING500 (Table S3). Interestingly, RWR using whole network performed worse than direct overlap, indicating that the connections between aging and ARDs are better identified through examining specific pathways or subnetworks. We also tested a method of directly overlapping aging and disease genes on subnetworks defined by GOs and KEGGs (see Supplementary Methods). This method performed even worse than direct overlap (Table S6). To explore the impact of *β*, we calculated the AUCs with *β* varied from 0.1 to 0.9 with step size of 0.1, similar to Shi *et al*.^20^. Though AUCs changed with *β*, the overall performance was not particularly sensitive to this parameter (Table S4). Based on the optimal AUC value, we decided to use GeroNet_E4 with STRING500 as the reference PPI network and set *β* to 0.1 in the following analyses.

Another potential issue about GeroNet is the redundancy among different biological processes, which is particular true in the case of Gene Ontology terms due to the special relational structures among the terms. To evaluate the impact of such redundancy, we implemented a greedy method to remove the overlapping GO terms and KEGG pathways (see Supplementary Methods). The performance of GeroNet on non-overlapping biological processes was shown in Dataset S10. It is clear that removing a few replicated or highly similar GO terms and KEGG pathways has very little impact on the overall results.

### Aging-disease subnetwork connections identified by GeroNet

We considered eMNs containing at least 5 aging genes and 5 disease genes since outputs from RWR are more robust with input of large number of seed genes. 149 (out of 277) disease/trait categories met this criteria in at least one eMNs among 3,581 unique eMNs. The full list of eMNs that show significant connections between disease and aging is provided in Dataset S3 (FDR < 0.05).

As shown in Fig. 2, the overall performance of GeroNet to rank diseases according to their association with aging is very good (AUROC = 0.84). The rank list of all 149 diseases/traits is provided in Dataset S4 and the top 30 diseases/traits are listed in Table 1. As can be seen in Table 1, “insulin-like growth factors” is ranked at the first place, which is probably the best known signalling pathway involved in aging and is highly conserved in multiple model organisms^21^. Followed in the list are various types of cancers or cancer-related traits (13 out of the top 30). The close connection between caner and aging has been well recognized. For example, according to Cancer Research UK (http://www.cancerresearchuk.org/about-cancer/type/lung-cancer/), lung cancer incidence is strongly related to age and almost nine in ten cases were diagnosed in individuals aged over 60 between 2010 and 2012 in the United Kingdom. Similarly, the incidence of melanoma in men aged 65 or older increases about eight fold compared with the men aged between 20 and 44 in 1991^22^. The incidences of many other cancers like bladder cancer, prostate cancer, leukaemias, and breast cancer also increase exponentially with age after maturity^23^. A popular hypothesis for aging-cancer association is that aged cells have accumulated more tumorigenic mutations over their lifespan compared to young cells. This seems to be supported by our findings as well. Based on their recurrences in the 13 cancers or cancer related traits, “intrinsic apoptotic signalling pathway in response to DNA damage” and “regulation of apoptotic signalling pathways” showed up in 9 and 10 cancers respectively and were ranked at top as shown in Table S5. Other top recurring modules include “signal transduction by p53 class mediator” and cell cycle related modules.

In addition to many cancers, AD, T2D, and myocardial infarction are also ranked as the top ARDs shown in Table 1. The incidence rate of AD begins to increase exponentially starting from around 65^24^ and the prevalence in US is about 9.7% for people over 70 years old^25^. According to the statistics from US centres for disease control and prevention (CDC) and a recent study in the United Kingdom^26^, the incidence of T2D begins to increase around 35–45, and peaks around 70 in both male and female. Similarly, as reported from US CDC, the risk of developing heart disease for men starts to climb at about age 45, when 1 out of every 100 men develop signs of heart disease; by age 55, the risk has doubled to about 2.1 out every 100 men; and it continues to increase until, by age 85, about 7.4 out of every 100 men have cardiovascular disease.

As supported by numerous epidemiological studies, GeroNet is effective in ranking well recognized ARDs to the top of the list. However, it is worth noting that a few ARDs were ranked near the bottom of the list. For example, atrial fibrillation, nephropathy, and inflammation biomarkers were ranked at 141^st^, 119^th^, and 112^nd^ out of 149 diseases (Dataset S4), even though they are supported by literatures as ARDs^27^,^28^,^29^. Such ranking errors could be caused by incompleteness or errors in our disease or aging gene lists, errors in the PPI network, or by the GeroNet algorithm itself. In addition, several diseases/traits ranked at the top of the list by GeroNet were not annotated as ARDs. Lipoprotein-associated phospholipase A2 activity and mass, menopause (age at onset), red blood cell count, and hypertriglyceridemia are just a few examples. Although they could be false positive predictions made by GeroNet, some evidence can be found to suggest that at least some of them could actually be age-related. For example, triglyceride levels often increase with age^30^.

### ARDs and non-ARDs show significant different interaction patterns with aging genes in modularized networks

By examining gene-gene interactions within modularized networks, we found that the disease-aging interaction patterns are very different between ARDs and non-ARDs in some of the subnetworks. We take AD and bipolar disorder (BD) as examples, which are both neurological diseases but show very distinct ages of onset. BD often occurs before 35^31^ while AD often occurs after 65^32^. AD significantly interacts with aging (FDR < 0.05) in 563 out of 797 total mapped modularized networks with a geometric mean of FDRs of 5.55 × 10^−4^. On the other hand, BD genes only map to 7 subnetworks and the interaction with aging is not significant in any of the 7 subnetworks, with a geometric mean of FDRs of 0.34 (Dataset S4 and S5). There are 3 modularized networks shared by both AD and BD, namely “GO0010959_regulation of metal ion transport”, “GO0005261_cation channel activity”, and “hsa04728_Dopaminergic synapse”. We plotted the interactions between aging, AD and BD in the subnetwork of “GO0010959_regulation of metal ion transport” in Fig. 3. As shown in the figure, this subnetwork contains 5 genes associated to BD (*ADCY2, CACNB2, ANK3, CACNA1C*, and *PIK3C2A*), 6 genes associated to AD (*NOS3, APP, PTK2B, PSEN1, GRIN3B*, and *PSEN2*), and 60 aging genes. Three genes are common to AD and aging (*APP, PTK2B*, and *PSEN1*), while no genes are shared by BD and aging. There are intensive interactions between AD and aging genes (85 in total), while only 19 interactions are seen between BD and aging genes. This difference is highly significant (p-value < 0.001) based on a permutation test in which we randomized the label of disease and aging genes. This example suggests that non-ARDs can have much fewer interactions with aging genes in certain subnetworks, and such network features can help us to mechanistically understand and differentiate ARDs from non-ARDs.

### Subnetworks with strong aging-disease connections as identified by GeroNet

We evaluated the connections between aging and 149 diseases/traits over 3,051 unique functional subnetworks. Among all the 52,090 disease-subnetwork pairs being checked, 22,280 pairs (~42.8%) showed significant aging-disease association at FDR< = 0.05 (Dataset S3). This indicates that aging potentially has a very broad impact to a large number of biological sub-systems and many of them could be involved in the connections with diseases.

**Figure 4:**
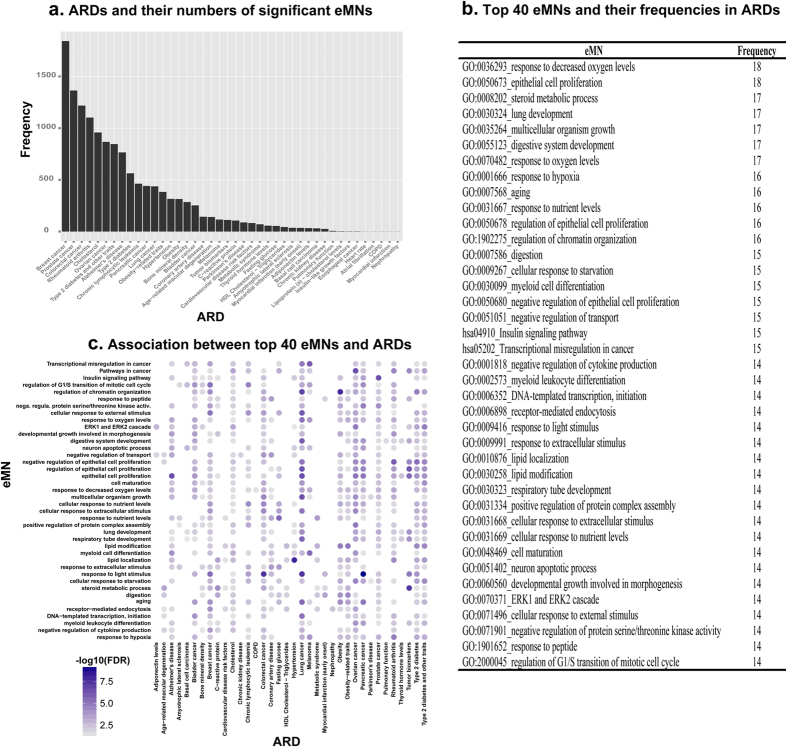
Connections between aging and ARDs in the top 40 common eMNs. (**a**) ARDs and their numbers of significant eMNS. (**b**) The top 40 most frequent eMNs and their frequencies in ARDs. (**c**) Association between the top 40 eMNs and ARDs.

To obtain a high level overview of the connections between aging and ARDs, we ask two questions: (1) for each disease, how many subnetworks are potentially involved in connecting with aging? and (2) are some subnetworks more frequently involved in mediating the connection between aging and diseases compared to others? We considered all the 22,280 significant subnetwork connections between aging and diseases. Out of the 149 diseases (43 ARDs and 106 non-ARDs as annotated based on literature), 106 diseases showed significant connection with aging in at least one eMN. Among these 106 diseases, 42 are ARDs and 64 are non-ARDs. This indicates that 98% of the ARDs have at least one subnetwork connection between aging and disease genes, while this fraction is only 60% for non-ARDs. This again supports that ARDs have more subnetwork connections with aging compared to non-ARDs in general, and these subnetwork connections identified by GeroNet are informative for differentiating ARDs vs. non-ARDs. We plotted the number of significant eMNs related to 42 ARDs in Fig. 4a. ARDs having extensive connections with aging in multiple subnetworks include many cancers (e.g., breast cancer, prostate cancer, and melanoma) and some non-cancer disease/traits (e.g., cholesterol, rheumatoid arthritis, T2D, obesity, and hypertension). On the other end of the spectrum, some ARDs are linked to aging through a relatively smaller number of pathways, such as heart rate and COPD. This result suggests that among ARDs, the connections with aging are potentially mediated by very different numbers of subnetworks.

On the other hand, different biological processes have different frequencies of being employed to connecting aging and diseases. We listed the top 40 most frequently involved eMNs across 42 ARDs in Fig. 4b. The hub eMNs encompass functions such as “response to decreased oxygen levels” and “cell cycle regulation”. For example, 18 ARDs (out of 43) were significantly associated with aging on the subnetwork related to “GO0036293_response to decreased oxygen levels”. These 18 ARDs included many cancers, AD, T2D and obesity (Fig. 4c). Decreased oxygen levels is known to associate with aging^29^. Hypoxia-Inducible Factor (HIF)-1 is activated in the body’s response to low oxygen concentrations and plays an integral role of shifting to anaerobic metabolism, promoting tumour survival and supporting inflammatory responses^33^. Hypoxia condition and its potential impact to diseases have been widely reported in cancers^32^, AD^34^, and diabetic conditions^35^. We plotted the significance of the connection with aging in top 40 most frequent subnetworks in Fig. 4c (ARDs that have no significant connections with aging in these top eMNs are not shown). Similarly to Fig. 4a, cancers, T2D and a few other diseases showed strong connections with aging in many of these eMNs.

Compared to many eMNs that are involved in mediating the connection between aging and multiple ARDs, a small number of eMNs (250 significant at FDR< = 0.05) were found to be disease specific (i.e., eMN in which only one disease has significant connection with aging) (Dataset S7). For example, subnetwork related to “GO1903792_negative regulation of anion transport” is significant only for T2D. Deacon *et al*. showed that anion transport is related to T2D^36^, meanwhile the involvement of anion transport in aging process has also been revealed^37^, indicating the potential role of anion transport in linking aging and T2D.

We present more detailed results for four ARDs, namely AD, prostate cancer, T2D, and Parkinson’s disease, while provide results for other diseases in the supplementary data. The top 10 eMNs mediating the interactions with aging and each of the four diseases are shown in Table 2. In particular, “GO0000302_response to reactive oxygen species” and “GO0030307_positive regulation of cell growth” were identified as significant modules for all the four diseases (Dataset S3). Reactive oxygen species (ROS) are known to damage DNA, RNA, and proteins, and are believed to have significant role in aging^38^. Their involvement in cancer^39^, AD^40^, Parkinson’s disease^41^, and T2D^42^ have been reported. Other oxidative and energy-related biological processes, e.g., “GO0022900_electron transport chain”, “GO0022904_respiratory electron transport chain”, and “GO0044455_mitochondrial membrane part” were also ranked at the top for these ARDs. One well established theories of aging is mitochondrial theory, which suggests that mitochondria is a main target of radical damage^43^. Electron transport chain deficiency has been found in Parkinson’s disease^44^, T2D^45^, AD^46^, and prostate cancer^47^. Our results are consistent with the hypotheses that electron transport chain and oxidative stress are among the major biological mechanisms connecting aging and various ARDs.

### Key connectors from subnetwork mediating aging-disease associations with specific biological functions

**Figure 5:**
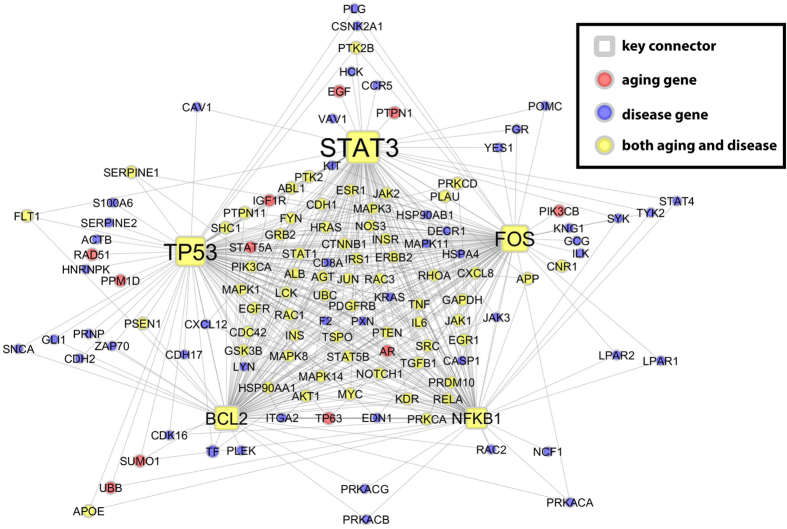
Key connectors of AD in extrinsic component of plasma membrane. We use node shape to denote key connectors: (1) square represents the top 5 key drivers; (2) circle represents expanded aging and disease genes. We use fill color to denote new (expanded) aging and disease information: (1) yellow represents the overlapping new aging and disease gene; (2) blue represents disease but not aging gene; and (3) red denotes aging but not disease gene.

We inferred putative genes connecting aging and ARDs for selected eMNs using a key driver analysis (KDA)^48^. KDA has been proven to be effective in identifying causal genes in breast cancer^49^ and late onset Alzheimer’s disease^16^. For example, in a breast cancer study, Tran *et al*. showed that driver genes identified by KDA were enriched by about 7 fold for genes led to reduced tumor cell viability compared to random selection based on results from siRNA knock down experiment^49^. The KDA was originally designed for directed networks, in which “master regulators” were found based on the “causal” regulation as reflected by the network topology. Since our PPI network is undirected, the hub genes identified by KDA are not necessarily “drivers” but are better considered as key connectors (KC) of aging and diseases. Therefore, we consider key connector analysis (KCA) as a better term in our case. The detailed information of key connector analysis (KCA) is provided in the Methods section. Using AD as an example: AD and aging are connected most significantly in a subnetwork corresponding to “GO0019897_extrinsic component of plasma membrane” (Table 2). With RWR expansion, there were 85 aging genes and 157 disease genes and the number of overlap between them was 68, with the Fisher’s exact test p-value less than 2.2E-16. We analysed these 68 common genes using KCA. The top 5 key drivers are shown in Fig. 5. The most significant key connector genes are *STAT3* and *TP53* with FDRs of 9.96e-33 and 1.03e-31 respectively. *STAT3* is a GenAge gene but not in the curated AD gene list. Study has shown that Tyk2/STAT3 signalling mediates β-amyloid-induced neuronal cell death which implicates its function in Alzheimer’s disease^50^. In addition, the roles of *TP53* in AD and aging have also been extensively reported^51^,^52^.

### Validation of GeroNet by an independent study in AD

In addition to numerous literature supports, we also considered validation by experimental data. Zhang *et al*. performed an integrated analysis on 1,647 post-mortem brain gene expression samples from patients of late-onset Alzheimer’s disease (LOAD) and identified multiple coexpression modules associated with LOAD^16^. To examine the relationships between each gene module with LOAD neuropathological traits, they calculated principal components (PCs) for each module and computed module-trait correlation. Finally, they ranked the modules and provided a list of top 20 modules each contained more than 50 genes. We performed a pair-wise comparison between their top 20 modules with our top 20 (expanded) modules identified by GeroNet on AD. Specifically, we used the 19,003 protein coding genes as background genes. For each module pair, we performed a Fisher’s exact test on the overlap of their genes and calculated adjusted p-values using Benjamini Hochberg method. The adjusted p-values are shown in Fig. S1 and Dataset S8. Interestingly, all of our top 20 modules significantly overlapped with at least one module in their study (FDR< = 0.05) as shown in Fig. S1, which is significant with p-value 0.01 based on a permutation test (see details in Method). Meanwhile, 12 of their modules significantly overlapped with our modules. Their top module (module “Yellow” with functions of Immune & microglia) significantly overlap with 15 modules in our study. These results indicate that GeroNet can identify highly relevant functions (e.g., immune related functions) associated with ARDs.

## Discussion

GeroNet provides a novel bioinformatics solution to systematically evaluate the complex connections between aging and diseases and sheds some new light on the following questions: (1) Diseases of different aging-dependency show different levels of network connections with aging genes; genes of ARDs often show more connections to aging genes in some subnetworks compared with non-ARDs; (2) Not all the pathways/subnetworks are equally important for mediating the connections between aging and ARDs; some subnetworks are common to many ARDs while some are rather disease specific; (3) A large number of potential key connections between aging and disease are identified and reported. Finally we show that our results can be validated by independent studies relying only on experimental data, confirming that meaningful biological insights can be achieved from GeroNet.

Compared to previous work, our study makes several significant improvements: First, we analyse at individual disease level instead of considering disease classes^15^. The study of individual diseases provides improved resolution in viewing the connections between aging and ARDs. Second, our modularized analysis reveals the biological contexts in which the aging genes and disease genes interact. Combined with KCA, our work generated a large number of testable hypotheses with potentials of developing new targets for translational studies.

It is of note that GeroNet could be used to infer connections between traits other than aging-disease. For example, it could be applied to studying the molecular associations between obesity and obesity-related diseases, between associated diseases to investigate their mechanisms of interactions, between environmental factors (e.g., smoking and drinking) and diseases, and so on. In the future, we plan to expand the application of GeroNet algorithm to to explore connections among different diseases and other biological/environmental processes.

On the other hand, GeroNet has its limitation and requires further improvement. First, GeroNet is based on the network reachability between aging genes and disease genes; although we have shown that this algorithm is globally valid and many top subnetworks and key genes are supported by literatures, the strong reachability may not necessarily imply a biological interaction between aging and disease. Therefore, some results are likely to be false positives, as indicated in Table 1 that some of the diseases ranked at the top were annotated as non-ARDs. Second, the aging genes in GenAge are collected from existing literatures and it is possible that existing publications are biased towards better studied aging genes. Other approaches of defining human aging genes in a more data-driven manner could help to reduce the biasness; for example, to call an aging gene based on gene expression data, such as Genotype-Tissue Expression (GTEx) and Multiple Tissue Human Expression Resource (MuTHER)^53^,^54^, or methylation data^55^. With many emerging interactions or regulations, such as eQTL (expression Quantitative Trait Loci)^56^, mQTL (methylation Quantitative Trait Loci)^57^, and microRNA-target regulation, we are facing many new opportunities to refine the algorithms of GeroNet and to build a more data-driven network that contains interactions of heterogeneous functional types to improve the identification of aging-ARD connections.

## Methods

### Compile aging and disease categories and genes by merging GWAS catalogue and OMIM

We downloaded aging gene list from GenAge Build 18^17^. The disease/trait associated genes were retrieved from NIH GWAS Catalog and OMIM (both were accessed on Dec 11, 2015)^58^. Some GWAS catalogue disease categories are closely related but named differently by different investigators. It will be helpful to merge the related groups of diseases so we have distinct disease groups while each group contains a relatively large number of genes. For that purpose, we applied a hierarchical clustering of the diseases, in which the distance between two diseases was defined as 1–*j*, where *j* is the Jaccard index of the two disease associated gene sets. We then manually merged diseases with similar definitions, e.g., uric acid levels and urate levels based on the tree structure. Similarly, we manually merged genes in GWAS and OMIM based on disease names. We eventually derived a list of 277 disease/trait categories and the full list is provided in Dataset S1.

### Literature mining for aging-disease association annotation

To annotate if a disease is age-related or not, we adopted a simple literature based text mining approach. Specifically, we ranked diseases/traits based on their Jaccard indices with the term “ageing” in PubMed abstracts published from 2008 to 2015. The PubMed abstracts containing the term “ageing” from 2008 to 2015 were retrieved using Entrez Programming Utilities (http://eutils.ncbi.nlm.nih.gov/entrez/eutils/esearch.fcgi?db=pubmed&term=[ageing]+ AND+2008:2015[pdat]&retmax=999999). We used the term “ageing” since it corresponds to “aging”[MeSH Terms] OR “aging”[All Fields] OR “ageing”[All Fields] in PubMed, which is a superset of the term “aging”.

The co-occurrence of a disease and aging was evaluated by the following equation


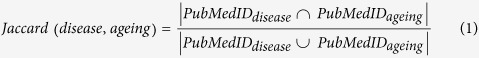


where 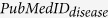 and 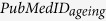 were the PubMed IDs containing the disease name and the term “ageing”, respectively. We then manually curated the ARD list, in which a disease/trait is annotated as being age-related if we identified strong literature evidence (see Dataset S2).

### Collection of reference networks

The reference PPI networks were obtained from Human Protein Reference Database (HPRD, Release 9)^18^ and Search Tool for the Retrieval of Interacting Genes/Proteins (STRING, Version 10)^19^. Six confidence levels (from 400 to 900 with step size of 100) of PPIs provided by STRING were considered separately, and were denoted as STRING400, STRING500, …, and STRING900. The summary statistics of proteins and interactions in the seven networks are listed in Table S1. Mathematically, we use a graph *G *= (*V, E*) to denote the reference PPI network comprised of a set of proteins *V* and a set of interactions *E*. Let ***A***_*n*×*n*_ be the adjacency matrix of ***G***, where *n* is the number of proteins. Clearly, the entry of *A* at row *i* and column *j* is 1 if protein *i* interacts with protein *j*; otherwise it is 0. The adjacency matrix ***A*** is column-wise normalized as


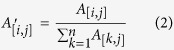


### Collection of GO terms and KEGG pathways

GO terms and KEGG pathways were downloaded from publisher’s websites^59^,^60^ on Dec 13, 2015. We consider GO BPs or KEGG pathways containing 30 to 500 genes, and ignore either very small or overly large functional gene sets.

### GeroNet analysis for aging-disease association

GeroNet first maps each selected GO BP or KEGG pathway onto the reference PPI network *G* to define a modularized network (MN). Specifically, a MN is a subnetwork of *G* spanning by all genes in a specific GO BP or KEGG pathway, which is further expanded by a random walk with restart on *G* to generate an expanded modularized network (eMN).

#### Random walk with restart

The random walker starts at a set of seed genes, which could be all genes in a MN (e.g., to derive the expanded MN), disease genes, or aging genes. The initial state *P*_0_ is formulated as a column vector 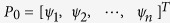, where 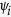 is set to 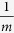 for the *m* seed genes and 0 for other genes in the network. It then randomly visits adjacent genes in every tick of time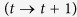. The state probabilities 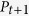 at time 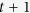 is calculated as following


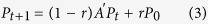


where *P*_*t*_ is the state probabilities at time *t, r* is the restart probability (i.e., starting from the seed genes again) and it is set to 0.7 as suggested by multiple previous studies^20^. This process is repeated until a steady-state is reached when the difference between two steps is smaller than 1e-6 as used by previous studies^61^. The steady-state probability of a gene represents how likely it will be visited from the seed genes and is used to rank the genes in *G* (genes with higher steady probabilities will be ranked closer to the top)^61^.

#### Expanded modularized network

Considering all genes contained in a modularized network as seeded genes, GeroNet performs RWR expansion on the reference network *G*, and ranks all genes in G based on their steady-state probabilities. The top 500 genes or the genes in the top N times of the size of original gene list (whichever is smaller) are considered as the nodes of an eMN with N fold expansion, while the edges between these nodes derived from the reference network *G* are used for edges in this eMN.

#### Aging-disease association on modularized network

We follow a method similar to GSEA (Gene Set Enrichment Analysis) to calculate a score indicating the reachability of two sets of genes on an eMN^31^. Specifically for each eMN, we first map the aging genes and genes associated to a disease onto it. By setting the mapped aging genes and disease genes as seeded genes, we perform two RWR expansions, which will rank all genes in the eMN according to disease genes and aging genes respectively. We first go through the sorted gene list of eMN according to disease genes, if we encounter a gene that is not an aging gene, 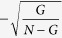 is added to the score, where *N* is the number of genes for the network, and *G* is the number of aging genes; otherwise, 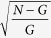 is added. This generates a curve like the one illustrated in Fig. 1b. The peak value of the curve is denoted as *ES*_1_ and the visited genes are defined as the “expanded disease genes” (e.g., 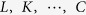 in Fig. 1b). Similarly, we go through the sorted gene list according to aging genes to calculate *ES*_2_ on disease genes and define the “expanded aging genes”. The enrichment score is defined as the weighted sum of scores


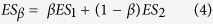


with 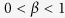. Here, *β* is a parameter to weight the relative importance of aging and disease genes. To determine the optimal value of *β*, we increase *β* from 0.1 to 0.9 with step size of 0.1; the value that results in optimal ARDs prediction is determined and used for later analyses.

The significance of *ES*_*β*_ is calculated based on a permutation test in which we permute aging genes in the eMN for 100 times to calculate the null distribution of enrichment scores (Fig. 1c). Base on this null distribution, observed *ES*_*β*_ is converted to a z-score statistic and a p-value is estimated and adjusted for multiple testing.

#### Rank diseases based on Aging-disease association

For any disease, the significance of its association with aging is defined as the geometric mean of the adjusted p-values for all eMNs. The diseases are then ranked based on their geometric mean p-values, and a disease with smaller p-value is considered more age-related.

### Evaluation Method 

To evaluate the performances of different methods and parameters, we compare their ranks of diseases (according to association with aging). Area Under the Curve (AUC) of Receiver Operating characteristic Curve (ROC) is calculated to see how reliable the rank is compared to the ARD annotation based on literatures.

### Key connector analysis

We adopted a previously established software package key driver analysis (KDA)^48^ to identify key connectors in PPI network. KDA was originally designed to identify “key regulators” in a directed regulatory network. When applied to undirected networks like PPI networks, we consider the key nodes as “key connectors” since they do not necessarily contain the directional information^48^. Such key connectors function more like a “hub” gene, instead of being considered as “master regulators”. Specifically, KDA takes a set of genes ***G*** and an undirected gene network ***N*** as inputs. It has two searching strategies namely dynamic neighbourhood search (DNS) and static neighbourhood search (SNS) for identifying key connectors. We adopted DNS in this study: (1) It first generates a subnetwork *N*_*G*_ consisting of all nodes in ***N***with no more than *L (L *= 2 in this study) steps away from the nodes in ***G***. (2) For each gene *g* in *N*_*G*_, DNS then searches for genes with distances no more than 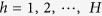 (*H *= 2 in this study) in *N*_*G*_. The set of genes (not including *g*) is denoted by 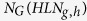. The Hypergeometric test is then used to calculate the enrichment between 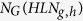 and ***G***with the genes in *N*_*G*_ as background for each *h*. The final enrichment p-value of each gene *g* is calculated as the minimum p-value across *h* layers. (3) The Bonferroni correction is performed to adjust for multiple testing and the genes with significant Bonferroni p-values (≤0.05) are outputted as key connectors.

### Significance of overlap between modules defined by Zhang’s study and GeroNet modules

We performed a permutation analysis to estimate the significance of module overlap result. In Zhang’s study, there were 22,331 genes (11,114 of them were unique) grouped into 64 modules (see “ Table S1-Module members” in ref. 16), among which 20 modules were considered as significant. We randomly reshuffled the 22,331 genes in the 64 modules (keeping the module sizes fixed) for 1,000 times. We then retrieved the 20 significant modules containing permutated genes and performed the same overlap enrichment analysis (as the previous section) with our top 20 modules. As a result, the mean number of overlapped modules between our top 20 modules and these permuted 20 modules in Zhang’s results among 1,000 runs was 17.30 with a standard deviation 1.24 (Dataset S9). The p-value of observing 20 significant overlapping modules is around 0.01 by assuming a one-tailed normal distributed observation, which indicates the significant overlap between our study and Zhang *et al*.’s work.

## Additional Information

**How to cite this article**: Yang, J. *et al*. Discover the network mechanisms underlying the connections between aging and age-related diseases. *Sci. Rep.* **6**, 32566; doi: 10.1038/srep32566 (2016).

## Supplementary Material

Supplementary Information

Supplementary Dataset 1

Supplementary Dataset 2

Supplementary Dataset 3

Supplementary Dataset 4

Supplementary Dataset 5

Supplementary Dataset 6

Supplementary Dataset 7

Supplementary Dataset 8

Supplementary Dataset 9

Supplementary Dataset 10

## Figures and Tables

**Table 1:** Top 30 most significant aging-disease associations inferred by GeroNet.

Disease	FDR	ARD	Disease	FDR	ARD
Insulin-like growth factors	1.61E-05	1	Type 2 diabetes	3.10E-03	1
Ovarian cancer	3.16E-05	1	Thyroid hormone levels	3.60E-03	1
Bladder cancer	7.47E-05	1	Chronic lymphocytic leukemia	3.76E-03	1
Lung cancer	1.14E-04	1	Lipoprotein-associated phospholipase A2 activity and mass	4.98E-03	0
Melanoma	3.40E-04	1	Menopause (age at onset)	4.99E-03	0
Alzheimer’s disease	5.55E-04	1	Myocardial infarction	5.11E-03	1
Type 2 diabetes and other traits	5.69E-04	1	Rheumatoid arthritis	5.75E-03	1
Tumor biomarkers	5.84E-04	1	Basal cell carcinoma	7.68E-03	1
Pancreatic cancer	6.52E-04	1	Red blood cell count	8.38E-03	0
Thyroid cancer	1.31E-03	0	Prostate cancer	9.03E-03	1
Acute lymphoblastic leukemia	1.53E-03	0	Obesity-related traits	9.29E-03	1
Breast cancer	1.73E-03	1	Adiponectin levels	1.04E-02	1
Myocardial infarction (early onset)	2.13E-03	1	Cholesterol	1.12E-02	1
Colorectal cancer	2.50E-03	1	Acne (severe)	1.12E-02	0
Inflammatory bowel disease	3.10E-03	0	Obesity	1.27E-02	1

**Table 2:** Function of the top 10 subnetworks for AD, prostate cancer, Parkinson’s disease, and T2D.

Alzheimer’s disease	FDR	Prostate cancer	FDR
GO0019897_extrinsic component plasma membrane	2.9E-15	GO0045639_positive regulation myeloid cell differentiation	3.8E-08
GO0031331_positive regulation cell catabolic process	2.3E-12	GO0010564_regulation of cell cycle process	4.1E-08
GO0009260_ribonucleotide biosynthetic process	1.8E-10	hsa04910_Insulin signaling pathway	3.2E-07
GO1903364_positive regulation of cellular protein catabolic process	1.3E-09	hsa04012_ErbB signaling pathway	3.4E-07
GO0070374_positive regulate ERK1 & ERK2 cascade	1.9E-09	GO0016051_carbohydrate biosynthetic process	3.5E-07
GO0071396_cellular response to lipid	7.6E-09	GO0045766_positive regulation of angiogenesis	6.1E-07
GO0009165_nucleotide biosynthetic process	1.1E-08	GO0002763_positive regulation myeloid leukocyte differentiation	7.2E-07
GO1903426_regulation of reactiveoxygen species biosynthetic process	1.1E-08	GO0001936_regulation endothelial cell proliferation	1.3E-06
GO0050921_positive regulation chemotaxis	1.4E-08	GO0044042_glucan metabolic process	1.4E-06
GO0031344_regulation cell projection organization	1.4E-08	hsa04062_Chemokine signaling pathway	1.5E-06
**Parkinson’s disease**	**FDR**	**type 2 diabetes**	**FDR**
GO0044708_single-organism behavior	3.4E-08	GO0005125_cytokine activity	4.5E-10
GO0010506_regulation of autophagy	9.8E-08	GO0032943_mononuclear cell proliferation	1.9E-09
GO1903146_regulation mitochondrion degradation	5.7E-07	GO0046651_lymphocyte proliferation	3.9E-09
hsa05016_Huntington’s disease	2.1E-06	GO0042100_B cell proliferation	4.9E-09
hsa05010_Alzheimer’s disease	2.9E-06	GO0070665_positive regulation leukocyte proliferation	4.5E-08
GO0022900_electron transport chain	5.0E-06	GO0042471_ear morphogenesis	5.3E-08
GO0022904_respiratory electron transport chain	6.0E-06	GO0042472_inner ear morphogenesis	9.8E-08
hsa05012_Parkinson’s disease	3.1E-05	GO2000379_positive regulation of reactive oxygen species metabolic process	1.7E-07
GO0046128_purine ribonucleoside metabolic process	3.3E-05	GO2000147_positive regulation of cell motility	2.9E-07
GO0044455_mitochondrial membrane part	3.4E-05	GO0070661_leukocyte proliferation	3.2E-07
