# Supplementary material for: Discover the network mechanisms underlying the connections between aging and age-related diseases
Source: Sci Rep. 2016 Sep 1;6:32566. doi: 10.1038/srep32566 (PMC5007654; doi:10.1038/srep32566)
Supplement: Supplementary Information [file srep32566-s1.doc]

# Supplementary: Discover the network mechanisms underlying the connections between aging and age-related diseases

**Jialiang Yang^1,2#^ , Tao Huang^1,2#¶^, Won-min Song^1,2^, Francesca Petralia^1,2^, Charles V Mobbs^3,4^, Bin Zhang^1,2^, Yong Zhao^1,2^, Eric E Schadt^1,2^, Jun Zhu^1,2^ and Zhidong Tu^1,2*^**

^1^ Institute of Genomics and Multiscale Biology, Icahn School of Medicine at Mount Sinai, NY, 10029, USA

^2^ Department of Genetics and Genomic Sciences, Icahn School of Medicine at Mount Sinai , NY, 10029, USA

^3^ Department of Neuroscience, Icahn School of Medicine at Mount Sinai, NY, 10029, USA

^4^ Department of Geriatrics and Palliative Medicine, Icahn School of Medicine at Mount Sinai, NY, 10029, USA

¶ Current: Institute of Health Sciences, Shanghai Institutes for Biological Sciences, Chinese Academy of Sciences, Shanghai 200031, People's Republic of China

^#^these authors contributed equally to this study

^*^corresponding author: Zhidong Tu, Department of Genetics and Genomic Sciences, Icahn School of Medicine at Mount Sinai, NY, 10029, USA. Tel: 212-659-8508, e-mail: [zhidong.tu@mssm.edu](mailto:zhidong.tu@mssm.edu)**.**

**Running title**: Identify network connections between aging and age-related diseases

**Supplementary Methods**

**Directly overlapping aging and disease genes on subnetworks defined by GOs and KEGGs**

For each subnetwork (defined by a GO or KEGG term), we first mapped the aging and disease genes onto it, we then calculated the Jaccard index of the mapped aging and disease gene sets. We permutated the mapped disease genes (among all the genes in the subnetwork) and calculated the Jaccard index (with the mapped aging genes) for each permuted disease gene set. Based on the permutation runs, we estimated a p-value of the overlapping between aging and disease genes (on the subnetwork). We then followed similar procedures as used by GeroNet to calculate the FDR, the geometric mean of FDRs (across all subnetworks), and to rank diseases. We tested HPRD and STRING with confidence score cut-offs from 400 to 900. The AUCs were listed in Table S6. As can be seen, the AUCs are significantly worse than GeroNet.”

**Remove redundancy among different biological processes**

For each disease/trait, we first ranked the subnetworks (generated by GOs and KEGGs) according to their adjusted p-values such that the networks with small p-values ranked in the front. We then filtered out overlapping subnetworks by searching the sorted subnetworks: (1) we kept the first subnetwork; (2) we then calculated the Jaccard index *J* between the nodes of the first and second networks. If *J* is smaller than a predefined the threshold *J*_T_ (*J*_T_ = 0.1, 0.2, … , 0.9), we considered the two networks to be non-overlapping and kept the second the network, otherwise we removed the second network. (3) Suppose we had *m* networks kept in the first *k* round of checking, we calculated the Jaccard indices of the k+1^th^ network with all *m* networks. If all indices are smaller than *J*_T_, we keep the network, otherwise we remove the k+1^th^ network.

We than calculated the geometric mean of the adjusted p-values only on the remaining non-redundant networks and ranked the diseases based on this value. The AUCs are listed in Dataset S10. Since the best AUC for GeroNet is 0.84, GeroNet with non-overlapping GOs and KEGGs is slightly worse than GeroNet. It is clear that removing a few replicated or highly similar GO terms and KEGG pathways has very little impact on the overall results.

**Supplementary Figures**

**Fig. S1- Comparing top 20 modules associated with Alzheimer’s disease identified by Zhang et al. and our study**

**
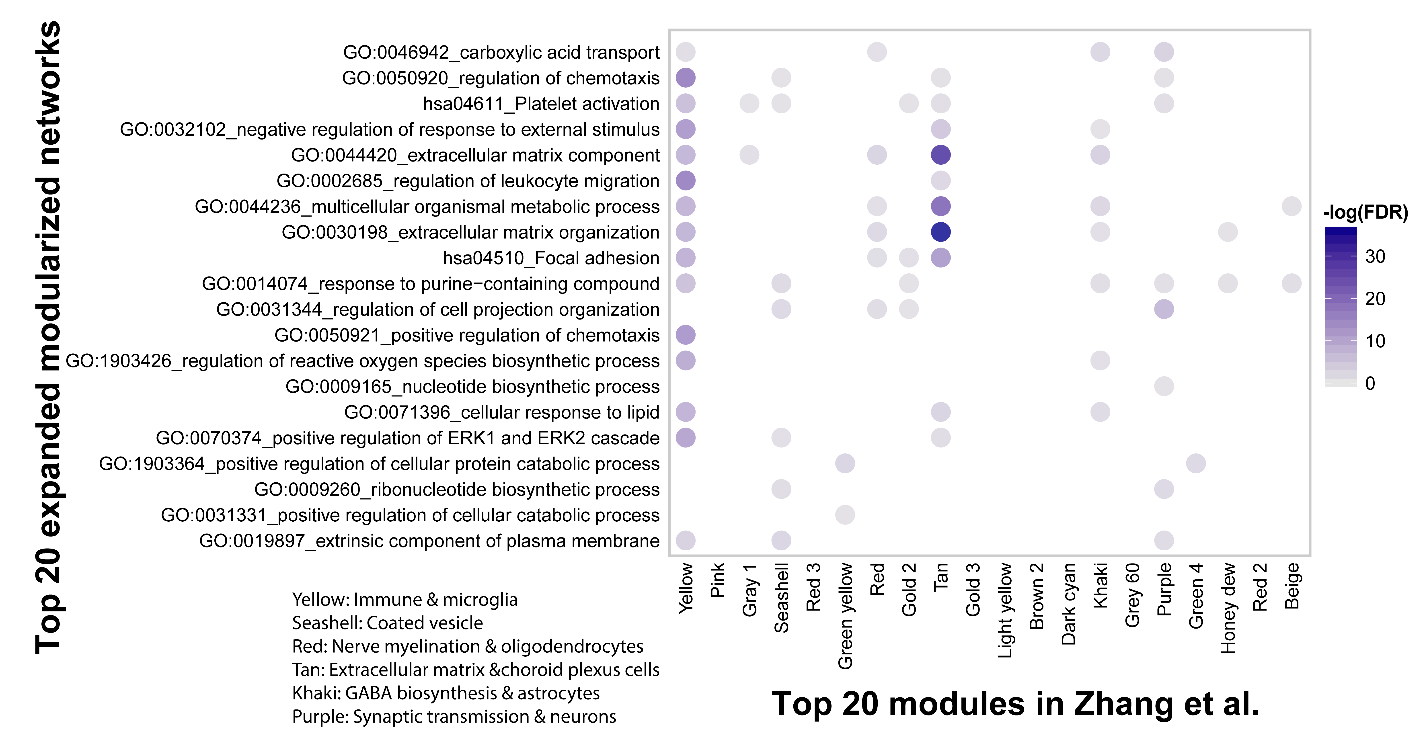
**

**Supplementary Tables**

**Table S1 - The statistics of nine** reference networks

| **Network** | **Number of protein** | **Number of interaction** |
| --- | --- | --- |
| HPRD | 9,465 | 37,039 |
| STRING400 | 17,862 | 690,097 |
| STRING500 | 17,473 | 500,362 |
| STRING600 | 16,178 | 374,520 |
| STRING700 | 14,782 | 302,469 |
| STRING800 | 13,119 | 257,239 |
| STRING900 | 10,687 | 206,674 |

*STRING400, STRING500,
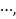
 and STRING900 indicate STRING PPI network with the interaction confidence score 0.4, 0.5,
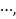
 and 0.9 respectively.

**Table S2 - Age-related diseases by literature mining**

| **Disease/Trait Name** | **Jaccard Ind** | **Disease/Trait Name** | **Jaccard Ind** |
| --- | --- | --- | --- |
| Alzheimer's disease | 5.99E-02 | Coronary artery disease | 4.55E-03 |
| Cardiovascular disease risk factors | 1.86E-02 | Chronic obstructive pulmonary disease | 4.24E-03 |
| Obesity | 1.84E-02 | Lung cancer | 4.01E-03 |
| Hypertension | 1.68E-02 | Tumor biomarkers | 3.88E-03 |
| Parkinson's disease | 1.52E-02 | Atrial fibrillation | 3.80E-03 |
| Cholesterol | 1.15E-02 | Colorectal cancer | 3.44E-03 |
| Bone mineral density | 1.14E-02 | Hippocampal atrophy | 3.43E-03 |
| Alzheimer's disease (cognitive decline) | 1.09E-02 | Proinsulin levels | 3.10E-03 |
| Type 2 diabetes | 1.08E-02 | Melanoma | 2.56E-03 |
| Heart rate | 9.13E-03 | HDL Cholesterol - Triglycerides | 2.11E-03 |
| Telomere length | 8.98E-03 | Rheumatoid arthritis | 2.03E-03 |
| Metabolic syndrome | 8.93E-03 | Adiponectin levels | 1.73E-03 |
| Pulmonary function | 8.73E-03 | Hepatocellular carcinoma | 1.58E-03 |
| Nephropathy | 7.83E-03 | Ovarian cancer | 1.53E-03 |
| Insulin-like growth factors | 7.36E-03 | Thyroid hormone levels | 1.23E-03 |
| Chronic kidney disease | 6.89E-03 | Pancreatic cancer | 1.21E-03 |
| Adiposity | 5.97E-03 | Bladder cancer | 8.50E-04 |
| C-reactive protein | 5.81E-03 | Multiple myeloma | 5.56E-04 |
| Age-related macular degeneration | 5.40E-03 | Basal cell carcinoma | 5.25E-04 |
| Stroke (ischemic) | 5.16E-03 | Esophageal cancer | 5.16E-04 |
| Myocardial infarction | 5.06E-03 | Chronic lymphocytic leukemia | 5.12E-04 |
| Fasting glucose | 4.92E-03 | Obesity-related traits | 3.01E-04 |
| Brain connectivity | 4.80E-03 | Lipoprotein (a) - cholesterol levels | 1.10E-04 |
| Breast cancer | 4.68E-03 | Myocardial infarction (early onset) | 8.05E-05 |
| Inflammatory biomarkers | 4.64E-03 | Type 2 diabetes and other traits | 5.15E-05 |
| Prostate cancer | 4.61E-03 | Laryngeal squamous cell carcinoma | 7.20E-06 |
| Amyotrophic lateral sclerosis | 4.60E-03 |  |  |

**Table S3 - The AUCs of aging-disease associations based on direct overlap, whole network, GeroNet_E1, and GeroNet_E4**

| **Network** | **Direct overlap** | **Whole network** | **GeroNet_E1** | **GeroNet_E4** |
| --- | --- | --- | --- | --- |
| HPRD | 0.77 | 0.59* | 0.73 | 0.78 |
| STRING400 | 0.77 | 0.72 | 0.76 | 0.81 |
| STRING500 | 0.77 | 0.70 | 0.78 | 0.84** |
| STRING600 | 0.77 | 0.70 | 0.79 | 0.81 |
| STRING700 | 0.77 | 0.69 | 0.79 | 0.78 |
| STRING800 | 0.77 | 0.69 | 0.77 | 0.77 |
| STRING900 | 0.77 | 0.67 | 0.76 | 0.75 |

* The AUC listed was the best AUC for
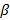
 from 0.1 to 0.9 with interval of 0.1

** The greatest AUC was based on geometric mean p value of sub-networks on STRING500 and
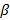
 0.1.

**Table S4 - The AUCs of aging-disease associations by GeroNet_E4 on STRING500 for different** $\beta\text{ }$

| 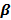 | **AUC** | 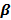 | **AUC** | 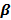 | **AUC** |
| --- | --- | --- | --- | --- | --- |
| 0.1 | 0.84 | 0.4 | 0.80 | 0.7 | 0.79 |
| 0.2 | 0.83 | 0.5 | 0.80 | 0.8 | 0.78 |
| 0.3 | 0.82 | 0.6 | 0.79 | 0.9 | 0.78 |

**Table S5 - Top 40 recurring functions for 13 cancers in Table 1**

| **Modularized Network** | **Cancer** | **Modularized Network** | **Cancer** |
| --- | --- | --- | --- |
| GO:2001233_regulation of apoptotic signaling pathway | 10 | GO:0042770_signal transduction in response to DNA damage | 9 |
| GO:0072331_signal transduction by p53 class mediator | 10 | GO:0007568_aging | 9 |
| hsa05202_Transcriptional misregulation in cancer | 10 | hsa05200_Pathways in cancer | 9 |
| GO:2000045_regulation of G1/S transition of mitotic cell cycle | 10 | GO:0071453_cellular response to oxygen levels | 9 |
| GO:1902807_negative regulation of cell cycle G1/S phase transition | 9 | GO:0070482_response to oxygen levels | 9 |
| GO:1902806_regulation of cell cycle G1/S phase transition | 9 | GO:0071456_cellular response to hypoxia | 9 |
| GO:0050673_epithelial cell proliferation | 9 | GO:0036294_cellular response to decreased oxygen levels | 9 |
| GO:0002573_myeloid leukocyte differentiation | 9 | GO:0001666_response to hypoxia | 9 |
| GO:0051321_meiotic cell cycle | 9 | GO:0036293_response to decreased oxygen levels | 9 |
| GO:0051402_neuron apoptotic process | 9 | GO:0030323_respiratory tube development | 9 |
| GO:0030099_myeloid cell differentiation | 9 | GO:0060541_respiratory system development | 9 |
| GO:0030183_B cell differentiation | 9 | GO:0035264_multicellular organism growth | 9 |
| GO:0050680_negative regulation of epithelial cell proliferation | 9 | GO:0007548_sex differentiation | 9 |
| GO:0009411_response to UV | 9 | GO:0031638_zymogen activation | 9 |
| GO:0071478_cellular response to radiation | 9 | GO:0030324_lung development | 9 |
| GO:1902275_regulation of chromatin organization | 9 | GO:0010950_positive regulation of endopeptidase activity | 9 |
| GO:0046660_female sex differentiation | 9 | GO:2001056_positive regulation of cysteine-type endopeptidase activity | 9 |
| GO:0006352_DNA-templated transcription, initiation | 9 | GO:0048732_gland development | 9 |
| GO:0051052_regulation of DNA metabolic process | 9 | GO:0071214_cellular response to abiotic stimulus | 9 |
| GO:0031625_ubiquitin protein ligase binding | 9 | GO:0044389_ubiquitin-like protein ligase binding | 9 |

**Table S6. The AUCs of direct overlapping on subnetworks defined** by GOs and KEGG pathways

| **network** | **AUC** |
| --- | --- |
| HPRD | 0.674 |
| STRING.cut0.400 | 0.666 |
| STRING.cut0.500 | 0.680 |
| STRING.cut0.600 | 0.690 |
| STRING.cut0.700 | 0.668 |
| STRING.cut0.800 | 0.657 |
| STRING.cut0.900 | 0.672 |

**Supplementary Data (downloadable at** <http://research.mssm.edu/tulab/dataset/agingdisease.html>**)**

**Dataset S1 - Aging and disease associated genes used in this study**

**Dataset S2 - Association between aging and 277 diseases by literature mining**

**Dataset S3 -** **Aging-disease association in expanded modularized networks**

**Dataset S4 - The rank of 149 diseases and traits by GeroNet**

**Dataset S5 - Aging interaction pattern difference between AD and Bipolar Disorder**

**Dataset S6 - Enrichment of aging and disease genes after mapping to the reference network**

**Dataset S7 - ARD specific eMNs**

**Dataset S8 - Overlap with Zhang top 20**

**Dataset S9 - Permutation results on the overlapping between the top 20 modules identified by Zhang and our study**

**Dataset S10 - The AUCs of GeroNet with non-overlapping GOs and KEGG pathways**
